# A New and Inexpensive Pyranometer for the Visible Spectral Range

**DOI:** 10.3390/s90604615

**Published:** 2009-06-12

**Authors:** Miguel A. Martínez, José M. Andújar, Juan M. Enrique

**Affiliations:** DIESIA, Escuela Politécnica Superior de la Universidad de Huelva, Ctra. Palos de la Ftra.-Huelva s/n, 21819, Palos de la Ftra. Huelva, Spain; E-Mails: andujar@uhu.es (J.M.A.); juanm.enrique@diesia.uhu.es (J.M.E)

**Keywords:** calibration, data acquisition, photodiode, pyranometer, solar irradiance, thermopile, visible spectral range

## Abstract

This paper presents the design, construction and testing of a new photodiode-based pyranometer for the visible spectral range (approx. 400 to 750 nm), whose principal characteristics are: accuracy, ease of connection, immunity to noise, remote programming and operation, interior temperature regulation, cosine error minimisation and all this at a very low cost, tens of times lower than that of commercial thermopile-based devices. This new photodiode-based pyranometer overcomes traditional problems in this type of device and offers similar characteristics to those of thermopile-based pyranometers and, therefore, can be used in any installation where reliable measurement of solar irradiance is necessary, especially in those where cost is a deciding factor in the choice of a meter. This new pyranometer has been registered in the Spanish Patent and Trademark Office under the number P200703162.

## 1. Introduction

This paper presents the design, construction and testing of a new pyranometer for measuring global solar irradiance (W/m^2^) or global solar radiation flux density within the visible spectral range (approx. 400 to 750 nm). Although the sensing element is a silicon photodiode, the developed pyranometer presents some characteristics and features similar to those of pyranometers based on thermopiles [1] at a price which is tens of times lower. This new pyranometer also incorporates significant additional features in terms of connectivity, measuring and remote programming and operation. The presented pyranometer can be used in any installation where reliable measurement of solar irradiance is necessary, especially in those where cost may be a deciding factor in the choice of a meter.

Generically, a pyranometer is a device for measuring solar radiation on a normally flat surface, in a field of 180 degrees. Measurement of solar radiation per unit of surface (W/m^2^) is termed irradiance. Irradiance measurement requires, by definition, that the pyranometer's sensor's response to radiation varies with the cosine of the angle of incidence from a line vertical to the surface of the sensor. That is, the maximum response is obtained when the radiation reaches the sensor perpendicularly (the sun at its zenith), while no response is obtained when the sun is on the horizon (an angle of incidence of 90°) and the response is half of the maximum when the incident radiation is at 60°. Therefore, it can be deduced from the definition that a pyranometer must have a “directional” response or, as it is usually termed, a cosine response to emphasise the fact that its response must ideally be analogous to the cosine function. The difference between the pyranometer's real response and the ideal cosine response is termed cosine error [2,3].

Pyranometers are widely used in meteorology, climatology, agriculture [4], solar energy studies [5] and building physics. In photovoltaic solar installations they are normally mounted with the sensor surface in the plane of the panel. In spite of the interest in measuring solar radiation, the use of pyranometers is still not very widespread outside the field of research, probably due to their high cost.

The element that characterises a pyranometer is the sensor it uses, which may be thermal (thermopile) or photovoltaic. Photovoltaic sensors are a cheap alternative, whose only advantage in principle over thermopiles in measuring radiation, aside from their price, is their response speed. Thus, while photodiode-based pyranometers have a response time of around 10 μs [6], in those based on thermopiles, response time ranges between 1 and 10 s, thus making them less suitable for measuring very rapid changes in radiation.

The influence of temperature on pyranometer's measurement is also well known. Although this influence exists, it is lower in thermopile pyranometers [1,7-10] than in photodiode devices [11-14].

With regard to integrating a pyranometer into an instrumentation system (generally into any measuring device), there is a series of very important factors to take into consideration, namely: ease of connection, signal degradation due to the transmission process [15].

In order to achieve the objective proposed in this work, designing and building a photodiode-based pyranometer [16] with similar characteristics to those of a thermopile-based device, also incorporating significant connection, measuring and programming utilities [17], the authors have analysed and corrected both the defects mentioned in literature and those observed during the testing of various commercial units. That is, the pyranometer developed has the following original features:

1. Excellent cosine response guaranteed by both the level gauge (to guarantee horizontality), which is incorporated, and by the specifically designed solar radiation diffuser.
2. Insensitivity in measuring variations in ambient temperature: A control circuit keeps temperature constant in the interior of the device.
3. Its interior incorporates all necessary electronics for both conditioning, and controlling and communications, which minimises noise and the need for auxiliary electronics.
4. It can be connected directly to a standard instrumentation system (PC, weather station, etc.): For this, the developed pyranometer has an RS 485 full-duplex serial digital port incorporated (i.e., directly-transmitted and received signals are digital).
5. It is equipped with a specifically designed virtual instrument (VI) which allows acquiring and storing measurements in different formats, configuring the parameters of the pyranometer (internal temperature, communications, etc.), reprogramming, controlling the pyranometer via the INTERNET, etc. from a PC.
6. Connection features in the developed pyranometer are significant, both in terms of their quality (ease, robustness, immunity to noise, etc.) and the cost-saving involved in not having to transmit and condition analogue signals outside the device.
7. In order to avoid internal condensation due to the temperature and air-tightness of the device—which may degrade its electronic circuitry and steam up the lens of the photodiode sensor—the developed pyranometer is equipped with a hygroscopic-salts container.
8. The cost is tens of times cheaper than that of a thermopile-based pyranometer of similar quality (including all signal conditioning and transmission circuitry).

The present paper is laid out as follows: Section 2 shall detail all the elements and systems constituting the pyranometer; Section 3 is devoted to experimental calibration procedures, measurement of relative spectral response and calculation of cosine error; and, finally, Section 4 presents the conclusions drawn.

## 2. System description

**Figure 1.**
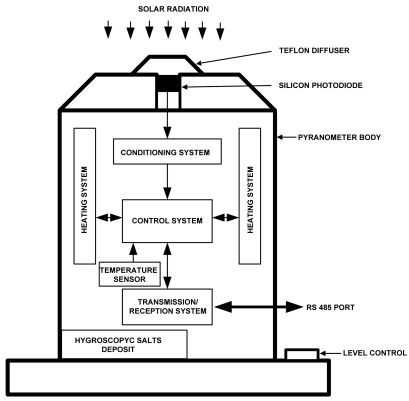
Pyranometer block diagram.

The developed pyranometer is shown outlined in its housing in Figure 1. The sensor element is a silicon diode, mounted on a plastic base, covered with a Teflon^™^ diffuser. The whole unit is placed on a base with a level control to ensure horizontality. In order to reduce undesirable effects of ambient temperature on measurement, the device is equipped with a temperature control system, so interior temperature remains constant while working, with an instruction issued by the control software and set by default at 40 °C (obviously this temperature may depend on the geographical area where the pyranometer is installed, therefore being adjustable by using software via the VI).

As it is well known [18], all the characteristics of photodiodes—parallel resistance, dark current, breakdown voltage, responsivity [19] and, to a lesser extent, transition capacitance—are affected by temperature changes.

The developed pyranometer generates an electrical signal proportional to the irradiance received in W/m^2^, which is subsequently converted inside the same device into digital format.

**Figure 2.**
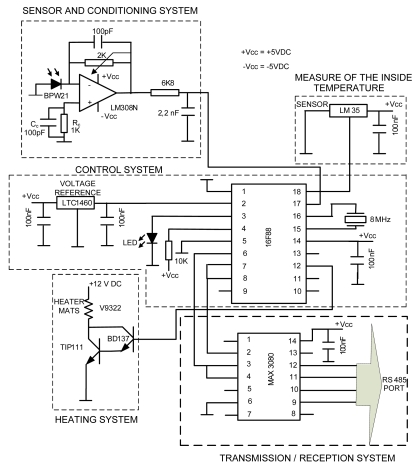
Pyranometer circuit diagram.

Each of the systems and elements with which the pyranometer is equipped will be described below. They are shown as a block diagram in Figure 1 and as a circuit diagram in Figure 2.

### 2.1. Radiation diffuser and pyranometer housing

**Figure 3.**
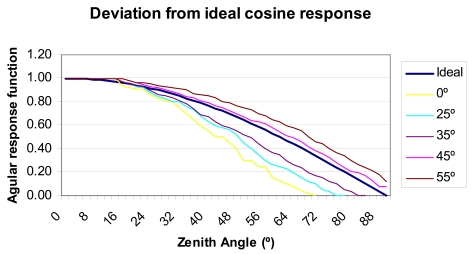
Deviation from ideal cosine response for different angles in the machined in the Teflon^™^ diffuser.

**Figure 4.**
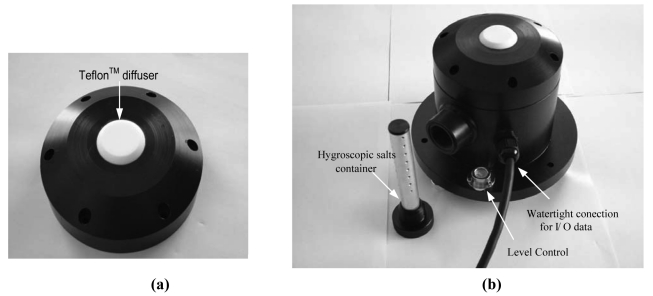
Developed pyranometer: (a) Detail of the diffuser in the pyranometer top. (b) Body of the pyranometer showing the container of hygroscopic salts and gauge level.

As a protective element for the sensor and at the same time a solar radiation diffuser (see Figure 1), a 5 mm thick Teflon^™^ cover has been designed and manufactured. Several thicknesses were tested for this piece (namely, 2, 3, 4 and 5 mm), although the one providing the best cosine response, with no loss incident radiation, was the 5-mm one. This piece is located just above the photodiode (see Figure 4.a). To a large extent this diffuser allows elimination of the cosine error [2,20,21]. Teflon has been used because it is a good diffuser and is also resistant to the elements and ultra-violet (UV) radiation [22,23], given its capability to diffuse transmitting lights nearly perfectly. Moreover, the optical properties of PTFE (Teflon^™^) remain constant over a wide range of wavelengths, from UV up to near infrared. Within this region, the relation of its regular transmittance to diffuse transmittance is negligibly small, so light transmitted through a diffuser radiates like Lambert's cosine law. Initially, a completely flat diffuser was designed and manufactured. However, after calibrating the pyranometer for the first time at the internationally homologated INTA (Spanish National Institute for Aerospace Technology) laboratory, it was changed for other machined ones at different angles. After a certain number of tests, it was decided that the best option was the one machined at 45°, since—as shown in Figure 3—the lowest cosine error was obtained at this angle.

Most commercial pyranometers use a glass dome which, apart from being more expensive than the Teflon^™^ diffuser used in this pyranometer, becomes affected by continuous solar radiation and traps higher amounts of dirt [24]. As a result, it must be replaced frequently to ensure device precision. The Teflon diffuser is housed together with the photodiode in the top of the pyranometer and is joined onto the rest of the body via six screws and a 50 mm O-ring, which allows complete air-tightness in the unit (see Figure 4a).

The pyranometer housing contains the photodiode and all the signal conditioning and distribution electronics. It is manufactured from a single piece of 10 mm thick black polyethylene, since polyethylene is a material which resists the elements very well and also shows excellent characteristics as a thermal insulator. Figure 4b shows the body of the developed pyranometer. It highlights the location of the watertight connection for data input/output, the level gauge (to achieve complete horizontality of the device) and the hygroscopic-salts container (to avoid condensation inside the pyranometer), which is accessible from the outside.

### 2.2. Sensor

The choice of pyranometer sensor element (photodiode) has required an exhaustive study of the commercial devices available, since it constitutes one of the key elements to being able to obtain better performance from the developed pyranometer. A photodiode was required with a response within the visible spectrum [25], a high value and as linear as possible.

In the search for the best sensor we worked in two different ways, on the one hand using the characteristics in the datasheets supplied by the manufacturers, and on the other carrying out real tests in our laboratories with the photodiodes selected.

The great variety of photodiodes analysed were classified into two types, namely, those which incorporate the conditioning circuit and those which do not. The former were rejected immediately, as they exhibited problems of saturation at high luminosity. As for the latter, the following were analysed: BPW21, OSD5-5T, OSD15-5T and S9219-01. In the datasheets for each photodiode the following characteristics were studied:

1. Radiant sensitive area (mm^2^) and spectral sensitivity (A/W). For a given irradiance (W/m^2^), these two characteristics allow the level of the signal provided by the photodiode to be known.
2. Noise equivalent Power (W/Hz^1/2^). Based on spectral sensitivity, this characteristic allows the noise-signal to be calculated. The signal produced by the photodiode divided by the noise-signal is its signal-to-noise ratio (SNR).
3. Price. The prices of the aforementioned photodiodes range from 7 to 20 Euros, with the cheapest being the BPW21. After the previous analysis, a practical test on the four photodiodes mentioned was carried out in the laboratory. For this, the following experiment was prepared to measure the voltage of each photodiode in short-circuit at different levels of irradiance.

**Figure 5.**
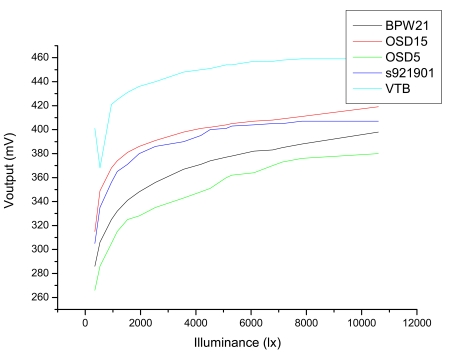
Curves of response of the different photodiodes analyzed, showing the voltage measured in the 0.1 Ω shunt depending on different illuminance levels.

The lamp used in the calibration laboratory is a quartz tungsten 1,000 W standard spectrum irradiance lamp (200A-H) with horizontal spiral filament, designated as S-1066, certified by NIST, and provided by Optronic Laboratories. Since instruments must be calibrated at the position in which they normally operate, using a lamp with horizontal filament becomes necessary [26]. This lamp, which was placed 50 cm from the photodiode, was used to emit the illuminance to be measured. Figure 5 shows the response of photodiodes (connected to a 0.1 Ω shunt element to carry out measurements in short-circuit) to different illuminances.

The response curves are certainly quite similar and the one showing the lowest response was that of OSD5-5T. VTB 101 was discarded because at low illuminance showed a strange behaviour, while it was also observed that its output was strongly dependant on temperature, since—insofar as lightness and, therefore, photodiode temperature increased—it showed irregular behaviour. Subsequently, we proceeded to complete a more detailed analysis on the selected photodiodes (namely, BPW21, OSD 5, OSD 15 and S9219-01). Table 1 was completed with the features provided by manufacturers.

The signal provided by each photodiode is the product of irradiance by active surface and by spectrum responsivity; therefore, from 1,000 W/m^2^ of irradiance onwards, the following is obtained for each photodiode (in the same order as that shown in Table 1):

(1) $$I_{p1}=1000\frac{\text{W}}{{\text{m}}^{2}}7.34\times 10^{-6}{\text{m}}^{2}0.34\frac{\text{A}}{\text{W}}=2.49\times 10^{-3}$$

(2) $$I_{p2}=1000\frac{\text{W}}{{\text{m}}^{2}}5\times 10^{-6}{\text{m}}^{2}0.15\frac{\text{A}}{\text{W}}=7.5\times 10^{-3}$$

(3) $$I_{p3}=1000\frac{\text{W}}{{\text{m}}^{2}}15\times 10^{-6}{\text{m}}^{2}0.21\frac{\text{A}}{\text{W}}=3.15\times 10^{-3}$$

(4) $$I_{p4}=1000\frac{\text{W}}{{\text{m}}^{2}}12.96\times 10^{-6}{\text{m}}^{2}0.22\frac{\text{A}}{\text{W}}=2.85\times 10^{-3}$$

As it might be expected, the greater the active surface is, the greater the generated signal is; however, noise also increases with surface. Therefore, signal-to-noise ratio (SNR) is a relative criterion which allows comparing them to select the most suitable. Assuming that measure circuits for each photodiode have the same bandwidth, noise for each photodiode is:

(5) $$I_{n1}=0.34\frac{\text{A}}{\text{W}}7.2\times 10^{-14}\frac{\text{W}}{{\text{Hz}}^{1/2}}=2.44\times 10^{-14}\frac{\text{A}}{{\text{Hz}}^{1/2}}$$

(6) $$I_{n2}=0.15\frac{\text{A}}{\text{W}}2.4\times 10^{-13}\frac{\text{W}}{{\text{Hz}}^{1/2}}=3.6\times 10^{-14}\frac{\text{A}}{{\text{Hz}}^{1/2}}$$

(7) $$I_{n3}=0.21\frac{\text{A}}{\text{W}}3\times 10^{-13}\frac{\text{W}}{{\text{Hz}}^{1/2}}=6.3\times 10^{-14}\frac{\text{A}}{{\text{Hz}}^{1/2}}$$

Note that noise produced by photodiode S-9219-01 is not shown, since the manufacturer provides no NEP (noise-equivalent power). Therefore, the SNR for each photodiode is:

(8) $$\text{SNR}(ph_{1})=\frac{I_{p1}}{I_{n1}}=\frac{2.49\times 10^{-3}\text{A}}{2.44\times 10^{-14}\text{A}/\sqrt{\text{Hz}}}=1.02\times 10^{11}$$

(9) $$\text{SNR}(ph_{2})=\frac{I_{p2}}{I_{n2}}=\frac{7.5\times 10^{-4}\text{A}}{3.6\times 10^{-14}\text{A}/\sqrt{\text{Hz}}}=2.08\times 10^{10}$$

(10) $$\text{SNR}(ph_{3})=\frac{I_{p3}}{I_{n3}}=\frac{3.15\times 10^{-3}\text{A}}{6.3\times 10^{-14}\text{A}/\sqrt{\text{Hz}}}=5\times 10^{10}$$

Note that BPW21 shows the best SNR. Consequently, bearing linearity, cost, photodiode active-surface and signal-to-noise ratio in mind, we decided that the most suitable photodiode for this application was BPW21.

### 2.3. Conditioning system

**Figure 6.**
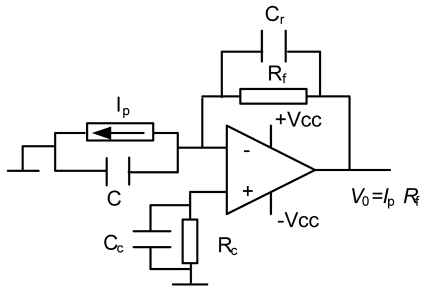
Transimpedance amplifier to condition the *I_p_* signal provided by the photodiode.

The transimpedance amplifier [27] shown in Figure 6, configured around the LM308N operational amplifier (OPAM), was used for signal conditioning from the photodiode (see Figure 2). In this circuit *Ip* is the photocurrent from the diode and *C* its parasitic capacitor. *C_c_*, *R_c_* and *C_r_* are compensation, correction and stabilisation elements respectively. Their value and function shall be seen later. Finally, *R_f_* is the feedback resistor which fixes the DC gain in the circuit, so the output from this is *V_0_* = *I_p_R_f_*. Note that the noise current in the photodiode has not been taken into consideration, since the BPW21 has an excellent SNR.

To calculate the value of *R_f_* a nominal irradiance of 1,000 W/m^2^ is used. For this, the BPW21 photodiode produces the photocurrent *I_p_* = 2.49 × 10^-3^ A. Therefore, as the maximum analogue input value accepted by the Analogue-to-Digital Converter (ADC) is 2.5 V, the value of *R_f_* is 1KΩ, which is implemented using a 2 KΩ multi-turn potentiometer to carry out precise adjustment.

In order to correct the DC error due to polarisation currents, a resistor (*R_c_*) is connected to the non-inverting input of the OPAM. This resistor has a detrimental effect in terms of noise [28], which is amplified; this is why a 100 pF compensation capacitor *C_c_* is connected in parallel with it. The parasitic capacitor on the photodiode BPW21, *C*, is 580 pF. This capacitor has to be taken into consideration, as it can influence the stability of the assembly (reducing its phase margin, and therefore, its relative stability). To improve the stability of the amplifier a capacitor *C_r_* is connected in parallel with the feedback resistor *R_f_*. Following the procedure laid down in the bibliography [27,28] it is calculated that an appropriate value for the capacitor is 100 pF. Finally, a low-pass filter is connected to the amplifier output (see Figure 2) set at the frequency of 10 Hz (*R* = 6K8 Ω and *C* = 2.2 μF). In this way the possible interference that could affect the ADC input is minimised.

### 2.4. Control system

**Figure 7.**
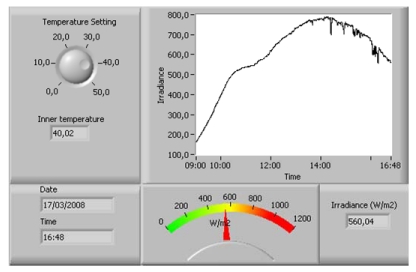
Virtual Instrument (VI) produced to control the pyranometer.

A PIC-type micro-controller (μc; manufactured by Microchip Technology Inc., see Figure 2) is used to control the entire pyranometer. The integrated circuit (IC) selected is 16F88, which incorporates an ADC. The ADC in the PIC acquires the conditioned analogue signal from the photodiode and converts it into digital format. Then, the digital signal generated is sent to the transmission system, made up of an RS 485 full-duplex serial bus converter: MAX3080. The PIC also maintains the inside of the pyranometer at a constant temperature. For this reason, it receives the signal from an analogue temperature sensor: LM35 (chosen for its stability and precision), fitted in the interior of the pyranometer housing. Based on measured temperature, the PIC executes the command to activate the heaters or not. This temperature can be adjusted by the user from a PC by using the VI (see Figure 7) for monitoring and controlling the pyranometer. Interior temperature is to be adjusted according to the climatic conditions in the area where the pyranometer is to be used. Specifically, in the area where tests were carried out (37° 12′ 02.70″N, 6° 55′ 10.19″W, elev. 19 m), temperature was adjusted to 40 °C. Finally, the PIC also controls disconnection of heaters automatically during the night with the aim of avoiding unnecessary energy consumption when the pyranometer is out of use.

### 2.5. Transmission/reception system

The transmission of bidirectional information between the pyranometer and the instrumentation system used (generally a PC, weather station or similar), is carried out in RS 485 full-duplex standard serial digital format [29]. This format is used over others because it allows connections between devices over a large distance (up to 1 Km without the use of signal repeaters), it is robust and very immune to noise. Control of communications is carried out by means of the IC MAX3080. This IC was chosen because it is completely programmable and allows all the communication parameters to be configured via software: transmission speed, parity, number of bits, full or half duplex, etc. TSB89 specifications were followed for the actual work of cabling [30]. The total number of wires to connect the developed pyranometer is eight [31]. That is, four for transmission of data and control-signals, three for power supply and one for the earth.

### 2.6. Heating system

Its job is to keep the temperature in the interior of the pyranometer constant at all times. Based on the operating temperature set by the user, the control system sends a signal to the thermostatisation system to activate the heaters until this temperature is reached. The heaters are heating meshes (circular elements) which run on 12 V with an approximate current consumption of 400 mA. Logically, the control signal from the PIC is not applied directly to the heaters, but to an electronic power stage, made up of BD137 and TIP 111 transistors (see Figure 2). The total power consumption of the device depends on the exterior temperature. However, the system is highly optimised, since the body of the pyranometer, made from 10 mm thick polyethylene, acts as an excellent thermal insulator. From the pyranometer control software, the user can select the minimum level of irradiance for the heating system to operate. This allows, for example, the pyranometer to stop working automatically at night and to start working, also automatically, by day. This utility allows optimisation of energy costs.

## 3. Results and Discussion

### 3.1. Calibration

Following construction of the pyranometer according to the specifications and methodology described, the next step was calibration of it [11,21,32-34]. With the aim of carrying out real comparisons of the effect the changes in ambient temperature produced in the response from the pyranometer, two identical pyranometers were constructed (the same geometry, sensor, diffuser and electronics) with a single difference: one had its interior temperature regulated and the other did not.

**Figure 8.**
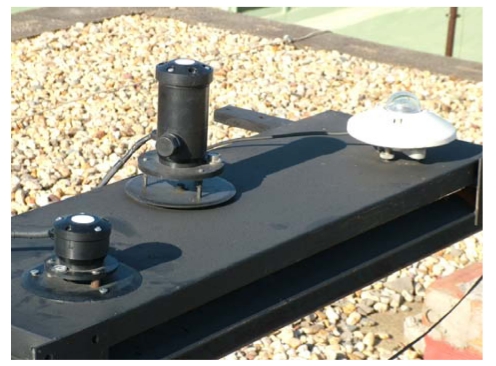
Location for calibrating the two pyranometers constructed, in relation to the standard pyranometer.

The process of calibrating the two pyranometers constructed to the elements was carried out following the ISO 9847 standard [35], by comparison with a standard pyranometer (SP), specifically the Kipp & Zonen CM21 which belongs to the *secondary standard class*, or the best. Pyranometers are standardised according to the ISO 9060 standard [36], which is also adopted by the World Meteorological Organisation (WMO). This standard discriminates three classes. The best is (confusingly) called *secondary standard*, the second best *first class* and the last one *second class*. Figure 8 shows the two pyranometers constructed together with the SP during the calibration process outside our laboratories (37° 12′ 02.70″N, 6° 55′ 10.19″W, elev. 19 m). The three pyranometers took irradiance measurements during 2008, with the aim of testing all the angles and intensity of solar radiation. The measurements were carried out every 10 s and were averaged every minute.

**Figure 9.**
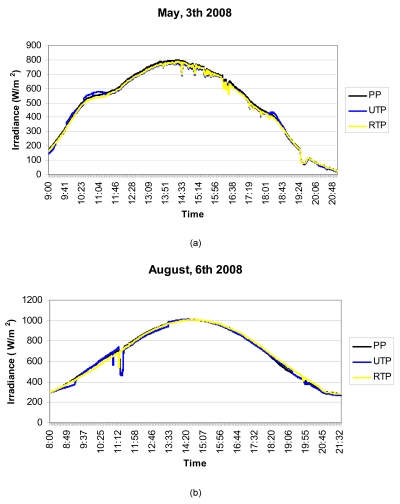

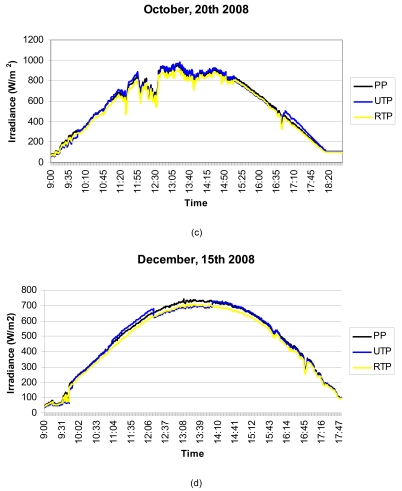
(a)-(d) Irradiance measurements from the three pyranometers (SP is the standard pyranometer, UTP is the unregulated temperature pyranometer and RTP the regulated temperature pyranometer) on four days spread throughout 2008.

Below are shown, by way of an example (see Figures 9a-d), the measurements obtained on four days spread throughout 2008. SP is the standard pyranometer; UTP is the unregulated temperature pyranometer and RTP the regulated temperature pyranometer.

**Figure 10.**
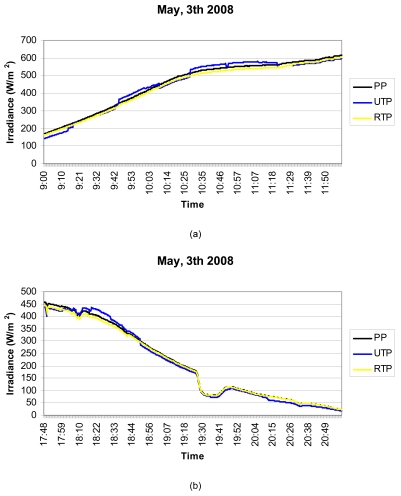
(a)-(b). Detail of the irradiance measurements from the 3^rd^ May 2008. In it the inferior performance of the UTP against the RTP can be observed.

Looking at Figures 9a-d, it can be observed that the RTP faithfully follows the SP curve at every time of day. Note how at times in the middle of the day when the ambient temperature is appreciably constant, the RTP and UTP reveal quite similar behaviour, however, early and late in the day, the UTP is much more sensitive to changes in ambient temperature. This can be observed in more detail in Figures 10a,b, where the early and late times of day respectively have been amplified from Figure 9a, corresponding to the curve for 3^rd^ May 2008. Note in the Figure how early in the day the UTP cannot follow the SP curve. Although its behaviour is worse than the RTP, in the last hours of the day (Figure10b), the UTP's deviation is not as pronounced as early in the day. This is due to the fact that the pyranometer housing accumulates the heat of the whole day and, therefore, its sensitivity to changes in ambient temperature, diminish when the sun sets.

Averaged throughout the year, the absolute error in relation to the standard pyranometer, is 2.31% for the UTP and 1.54% for the RTP. However, the UTP has very good response, and at least in geographical locations similar to those in this work, it is a serious option for applications which are not excessively demanding.

### 3.2. Relative Spectral Response (RSR)

**Figure 11.**
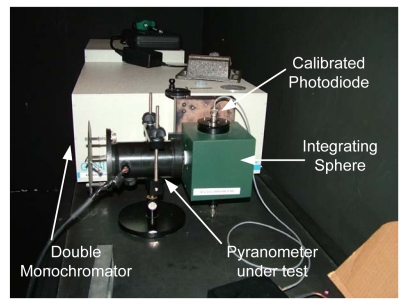
Photograph of the test carried out to determine the RSR (Relative Spectral Response) in the INTA (the Spanish Institute of Aerospace Technology) calibration laboratory in Huelva (Spain).

In order to measure the pyranometer's spectral response (see Figure 11), the device underwent testing at the INTA calibration laboratory. The calibration equipment consists of a Jobin Yvon Gemini 180 double monochromator, which has a 450 W xenon light source connected to it. This device has three slits whose apertures are located so that there is 2 nm Fwhm (Full width at half maximum) monochromatic light at the exit. An integrating sphere with two exits is placed at the double monochromator exit. A photodiode calibrated by the WRC (World Radiation Center) is placed at one of the sphere's exits and the pyranometer to be characterised is placed at the other. A scan is performed within the range 300 to 750 nm in 2 nm steps. In this way the RSR is determined for the developed pyranometer, using the photodiode calibrated by the WRC as a norm (see Figure 11).

**Figure 12.**
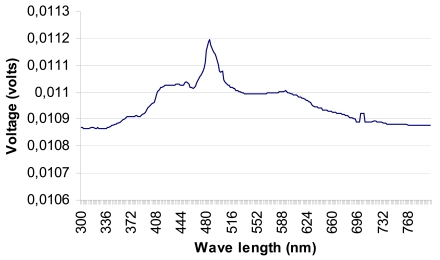
Spectral response curve obtained for the RTP pyranometer in the INTA calibration laboratory.

The results obtained are shown in Figure 12. Note that the pyranometer's spectral response is approximately between 300 and 750 nm. Although the maximum response is obtained around 500 nm, the pyranometer response curve is reasonably flat (± 0.15 mV over 0.01105 V, i.e., ± 1.36 %) throughout the visible light range (approx. 400 to 750 nm).

### 3.3. Calculation of the cosine error

**Figure 13.**
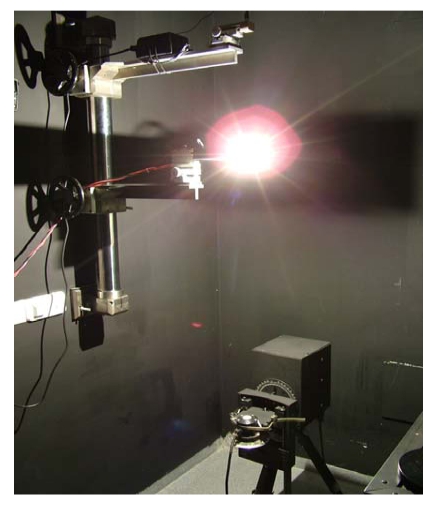
Photograph of the test carried out in the INTA calibration laboratory to calculate the cosine error of the pyranometer.

**Figure 14.**
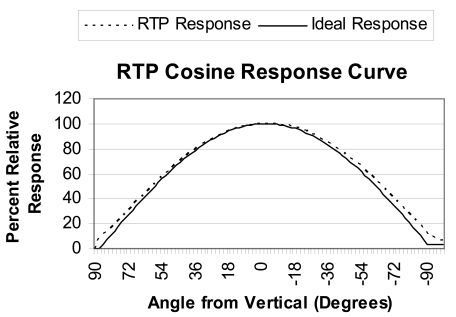
Ideal response and real measured response curves, both according to the angle of incidence

In order to calculate the cosine error for the pyranometer, the device underwent testing at the INTA calibration laboratory. The measuring procedure in the laboratory consists of rotating the pyranometer at a constant distance from a calibrated lamp, in such a way that the distance between the lamp and the photodiode is such that the filament can be considered a point source, so that there is no error introduced into the calibration. The equipment used is that photographed in Figure 13. In it can be seen the lamp at the top and the pyranometer undergoing calibration, mounted on a articulated robotic arm, which allows it to be turned between ± 90° with a high degree of precision. It is important to ensure that the lamp is perpendicular to the pyranometer sensor when the angle of incidence is zero above the rotating system, since, if this were not the case, false results might be produced at higher angles. A laser is used to perform this alignment. The automated system carries out an initial measurement at 0° and starts to turn one degree at a time taking measurements from - 90° to + 90. The results of the measured cosine response are shown in Figure 14. The Figure shows the measured cosine response for the RTP. That obtained for the UTP is analogous if the effect of temperature variation is isolated, i.e. if the temperature of the UTP is kept constant during the experiment.

The curve in Figure 14 represents the pyranometer's percentage response based on the radiation's angle of incidence from vertical. Since the pyranometer's ideal response must be analogous to the cosine of the radiation's angle of incidence, when this is perpendicular to the surface of the sensor (the sun at its zenith), the response must be maximum (100%). The response must be 50% when the angle of incidence is 60° and nil when the sun is on the horizon (angle of incidence of 90°).

Based on the data obtained from the calibration test, the cosine error [1], *δ_cos_*, is calculated by means of Equation (11):

(11) $$\delta_{\cos}=\frac{\frac{\left(U(z)+U(-z)\right)}{2}-\text{zero}(z)}{\left[\frac{U(0^{\text{o}})+U(0^{\text{o}})}{2}-\text{zero}(z)\right]\cos(z)}100\%$$

Where *U*(0^°^) is the pyranometer output voltage for normal incidence, *U*(*z*), the pyranometer output voltage for angles (*z*), and zero (*z*) the dark signal for angles (*z*).

The data obtained show that deviation from ideal response is less than 2% from 0 to ± 75 degrees and less than 3% from ± 75 to ± 85 degrees. If we take into consideration that the manufacturer [1] provides a deviation from ideal response of +/- 2% at 60° and +/- 6% at 80° for the SP (CM21) we can qualify the cosine response from the developed pyranometer as excellent.

## 4. Conclusions

This paper presents the design, construction and testing of a photodiode-based pyranometer for the visible spectrum. Tests carried out on the developed device show it can compete successfully with high-end commercially available pyranometers at a much lower price and with additional features in terms of connectivity, measurement and remote programming and operation. The newly developed pyranometer can be used in any installation where reliable measurement of solar irradiance is necessary, especially if cost becomes a deciding factor when choosing a pyranometer. This new pyranometer presented in this work brings together features which make it a very competitive alternative to what the market offers at present time. These features are: (1) Excellent cosine response. (2) Measurement insensitivity to variations in ambient temperature. (3) Incorporation of all necessary electronics in the device itself, both for conditioning and control and communications, which minimises noise and the need for auxiliary electronics. (4) It allows direct connection to a standard instrumentation system. (5) It is equipped with a specifically designed virtual instrument (VI) which allows acquiring and storing measurements in different formats, configuring the pyranometer's parameters (interior temperature, communications, etc.), reprogramming, control via Internet, etc. All the previous can be carried out from a PC. (6) The connection features included by the developed pyranometer are significant, both in terms of their quality (ease, robustness, immunity to noise, etc.) and cost-saving, since no signals must be transmitted and conditioned outside the device. (7) It incorporates a hygroscopic-salts container which prevents internal condensation due to the temperature and air-tightness of the device, which may degrade its electronic circuitry and steam up the lens of the photodiode sensor. (8) Its cost is several tens of times cheaper than a thermopile-based pyranometer of similar quality (including all signal conditioning and transmission circuitry). As shown in the present paper, an unregulated temperature version has been developed, manufactured and tested, which is even cheaper and perfectly suitable for not excessively demanding applications at a very low cost. The cost of the RTP—taking into account materials, labour and industrial profit—is around 70 Euros, that of the UTP being 55 Euros. This new pyranometer has been registered in the Spanish Patent and Trademark Office under the number P200703162.

## Figures and Tables

**Table 1.** Features of selected photodiodes.

**Photodiode**	**Sensitive area (mm^2^)**	**Responsivity (R_λ_) (A/W)**	**NEP (W/Hz^1/2^)**	**Price (Euros)**
BPW21 (ph1)	7.34	0.34	7.2 × 10^-14^	7.30
OSD 5-5T (ph2)	5	0.15	2.4 × 10^-13^	16.50
OSD 15-5T (ph3)	15	0.21	3 × 10^-13^	17.53
S9219-01 (ph4)	12.96	0.22	————	————
